# Hierarchical Semantic Transmission and Lyapunov-Optimized Online Scheduling for the Internet of Vehicles

**DOI:** 10.3390/s26092606

**Published:** 2026-04-23

**Authors:** Le Jiang, Yani Guo, Wenzhao Zhang, Penghao Wang, Shujun Han

**Affiliations:** 1 School of Cyberspace Security, Beijing University of Posts and Telecommunications, Beijing 100876, China; jiangledeyouxiang@bupt.edu.cn (L.J.); gyn@bupt.edu.cn (Y.G.); 18707731210@bupt.edu.cn (P.W.); 2 School of Information and Communication Engineering, Beijing University of Posts and Telecommunications, Beijing 100876, China; zhangwenzhao@bupt.edu.cn

**Keywords:** semantic communications, multi-vehicle collaborative perception, hierarchical transmission, online scheduling, Age of Information (AoI), lyapunov virtual queue

## Abstract

The inherent redundancy in vehicle sensor data, coupled with constrained onboard resources and stringent latency requirements, renders traditional bit-oriented transmission paradigms inefficient for autonomous-driving perception tasks. Semantic communication offers a promising direction by shifting the focus from bit-level fidelity to task-level information delivery. In this paper, we propose a unified framework that integrates hierarchical transmission and online scheduling for Internet of Vehicles (IoV)-oriented collaborative perception. The proposed hierarchy separates information into two complementary layers: a coarse metadata layer (object bounding boxes) for latency-critical awareness, and fine-grained visual semantics (multi-scale region-of-interest (ROI) patches) for perception-intensive tasks. We formulate an online scheduling problem that jointly exploits Age of Information (AoI) and Channel State Information (CSI) to dynamically decide what to transmit and at what fidelity under per-frame budget constraints. To address cross-scheme fairness, we report resource utilization under a fixed kbps/fps physical budget and evaluate robustness using a combination of a lightweight task-proxy metric and COCO-style Average Recall (AR100) under ROI-only evaluation. The hierarchical transmission architecture, combined with AoI awareness, reduces global semantic staleness by approximately 78%. The Lyapunov-based online scheduler enables intelligent, signal-to-noise ratio (SNR)-adaptive switching between coarse and fine semantic levels, ensuring robust perception under varying channel quality. Under strict physical-budget constraints and unreliable channel conditions, joint source-channel coding (JSCC) exhibits significantly stronger task robustness than conventional schemes: at 0 dB SNR, the task-proxy detection rate improves by nearly 47 percentage points over the uncoded baseline.

## 1. Introduction

In modern intelligent transportation systems (ITS), collaborative perception in the Internet of Vehicles (IoV) is widely regarded as a key technology for extending the safety boundary of autonomous driving and improving traffic efficiency. Through V2X communications, vehicles can share real-time environmental information captured by onboard sensors, thereby overcoming the blind spots of single-vehicle perception [1]. However, the traditional bit-oriented Shannon communication paradigm is gradually approaching its practical limit in highly dynamic vehicular networks [2]. In real IoV scenarios, communication links often experience low signal-to-noise ratio (SNR), frequent packet loss, limited or unavailable retransmission (ARQ), and stringent end-to-end latency constraints. Under these adverse physical conditions, conventional separation-based digital source and channel coding schemes such as JPEG and H.26x are highly susceptible to the well-known cliff effect [3], where task performance deteriorates abruptly once the channel quality falls below the design threshold.

In recent years, task-oriented semantic communications have emerged as a promising paradigm for overcoming these limitations [4,5]. The core idea of semantic communications is not to preserve the entire frame at the highest possible pixel fidelity, but to extract and deliver the most meaningful information for downstream intelligent tasks such as object detection and tracking under limited system budgets [2,4]. Deep joint source-channel coding (DeepJSCC) has already been shown to achieve graceful degradation in low-SNR regimes [3]. At the same time, object-centric or region-of-interest (ROI)-based semantic cropping has been widely explored to remove redundant background information and preserve more task-relevant semantics under constrained bandwidth [6,7].

Nevertheless, most existing studies address only one technical dimension in isolation and lack both dynamic resource orchestration over continuous time and system-level closed-loop validation. Three major challenges remain:

- Temporal semantic freshness is not explicitly modeled: Detection confidence alone cannot reflect the accumulated safety risk of targets such as pedestrians that remain occluded or outdated for a long time. The system should prioritize safety-critical objects that have not been updated for many frames [8,9], which motivates the introduction of AoI for temporal correction [10].
- CSI-unaware scheduling leads to resource mismatch: Without channel awareness, high-SNR conditions may waste bandwidth while low-SNR conditions may suffer severe distortion or even decoding failure.
- A fair comparison across coding regimes is often missing: Prior comparisons between analog-style semantic transmission and conventional digital coding usually fail to unify the physical meaning of channel occupancy.

To address these challenges, this paper presents a unified closed-loop framework that integrates hierarchical semantic transmission with online scheduling for collaborative perception in IoV. Unlike studies that rely solely on reconstruction metrics such as PSNR or MSE, we emphasize task-related proxy metrics and fair benchmarking under fixed kbps/fps budgets and non-ideal link conditions. While the individual ingredients (ROI-based perception, JSCC-style analog transmission, and Lyapunov-based resource allocation) have been explored in prior work, their system-level integration under strict per-frame constraints and their end-to-end task-centric validation remain less studied. The main contributions of this work are summarized as follows:

1. Hierarchical ROI representation for budgeted perception: We develop a two-layer transmission payload that separates perception into coarse-grained metadata (object bounding boxes, BBox) as a low-cost fallback and fine-grained visual semantics (multi-scale regions of interest, ROI patches), tailored to object-centric scheduling under strict per-frame budgets.
2. AoI/CSI-aware online scheduling with a virtual queue: We adopt a Lyapunov drift-plus-penalty (DPP) formulation with a virtual queue to stabilize the long-term bandwidth budget and instantiate an online scheduler that jointly exploits AoI and CSI to select BBox metadata and multi-mode ROI patches under a strict per-frame constraint, together with a lightweight greedy packing heuristic for real-time execution.
3. Fair physical-budget accounting and task-centric robustness metrics: To avoid biased cross-scheme accounting, we report resource utilization under a fixed kbps/fps physical budget and evaluate robustness using a combination of a lightweight task-proxy metric and COCO-style AR100 under ROI-only evaluation, together with Tx-normalized AR100 to isolate transmission-induced degradation under the same transmitted ROIs.
4. System-level integration and end-to-end validation: We conduct system-level ablations on continuous driving video streams to validate the roles of AoI, CSI awareness, and virtual-queue stabilization. We further provide robustness studies (budget sweep, SNR sweep, drop scan, and fading sanity) and a runtime profiling table to support real-time feasibility claims.

## 2. Related Work

### 2.1. Semantic Communications and JSCC for IoV

The primary goal of semantic communications is to extract and deliver the most task-relevant meaning to the receiver rather than approaching channel capacity on a bit-by-bit basis [2,4]. In collaborative perception for autonomous driving, the receiver is often a machine-vision model such as a YOLO detector, and therefore task usability, rather than pure pixel fidelity, becomes the most meaningful quality criterion. The DeepJSCC framework proposed by Bourtsoulatze et al. is a seminal work in this area. It departs from the conventional source-channel separation paradigm and directly maps raw images to continuous channel symbols in an end-to-end manner, exhibiting significantly smoother degradation than conventional digital coding in low-SNR conditions [3]. Building on this line of work, researchers have further explored CSI feedback, adaptive model selection, channel-blind robustness, attention enhancement, and lightweight semantic transmission [11,12,13,14,15,16], making JSCC increasingly suitable for time-varying IoV links and edge-constrained deployment.

At the same time, ROI-guided semantic transmission has attracted extensive attention. ROI-based semantic communication allocates heterogeneous bandwidth to semantically important regions and removes redundant background, thereby preserving more task-relevant information under constrained budgets [7,17,18]. In addition, cloud-edge collaborative video analytics frameworks [6] also reduce communication overhead by selectively transmitting visual content or features. However, most existing ROI-based semantic-communication studies assume static per-frame resource allocation and rarely model temporal continuity across frames or the evolution of object importance over time. As a result, they cannot guarantee sustained semantic freshness and consistency in continuous video streams.

It is also worth noting that traditional image/video coding has long supported layered or scalable representations (e.g., base layers and enhancement layers) that enable adaptive decoding at different qualities. Conceptually, this is related to our use of a low-cost fallback (BBox metadata) and higher-cost ROI patches. The key difference is that our system performs task-driven, object-centric mode selectionand online packing under a per-frame budget with explicit AoI/CSI awareness, and it directly evaluates the resulting semantics using downstream detection-oriented metrics rather than focusing only on reconstruction quality.

### 2.2. Age of Information (AoI) and Semantic Freshness

Age of Information (AoI) is defined as the time elapsed between the current instant and the generation time of the most recently received update at the destination. It is a fundamental metric for information freshness [10]. In the highly dynamic IoV environment, low transmission latency does not necessarily imply high semantic value. A low-latency packet carrying little task importance may contribute much less to system safety than a slightly delayed packet carrying critical target information. Accordingly, AoI-aware scheduling and resource-allocation strategies have been investigated for broadcast networks [19], vehicular resource management [8,20], and resource-constrained relaying and scheduling [9].

With the development of semantic communications, researchers have increasingly combined AoI with semantic importance and proposed concepts such as Age of Semantics (AoS) to characterize the temporal decay of semantic effectiveness more precisely [21]. This direction has shown early promise in UAV-assisted wireless networks [21] and IoV scenarios over error-prone channels [8]. In addition, joint optimization of AoI and queue management has demonstrated potential in V2V networks [22]. Building on these insights, this paper maintains a lightweight IoU-based tracker at the edge and fuses AoI with target-category priors to construct a dynamic scheduling score that is both theoretically meaningful and practically implementable, allowing the scheduler to prioritize targets that are both safety-critical and semantically stale under limited budgets.

### 2.3. Lyapunov Optimization and Online Resource Allocation

In IoV environments with limited resources and time-varying channels, bandwidth allocation is inherently a stochastic optimization problem with long-term average constraints. Lyapunov stochastic optimization addresses such problems by constructing a virtual queue to represent resource deficit and using the drift-plus-penalty (DPP) framework to transform an otherwise intractable long-term optimization problem into a sequence of tractable per-slot deterministic problems, thereby achieving asymptotically optimal long-term performance without requiring future channel knowledge [9,23,24]. This framework has been validated in scenarios including semantic resource allocation in D2D vehicular networks [23], adaptive semantic resource management [24], reinforcement-learning-based resource scheduling in vehicular networks [25], and semantic-aware task scheduling or offloading in vehicular edge computing [26].

However, most prior studies apply Lyapunov optimization to continuous action spaces such as power or bandwidth allocation, or combine it with deep reinforcement learning to address high-dimensional state spaces [27]. Only limited attention has been paid to discrete multi-mode selection problems with strict per-frame constraints, especially when online packing is required. In this paper, we combine the DPP framework with greedy online packing over multiple ROI patch modes. The resulting virtual-queue backpressure mechanism stabilizes the long-term budget across frames while balancing semantic reward and channel cost at each frame.

## 3. System Model and Accounting Metrics

### 3.1. End-to-End Pipeline

For each frame *t*, the system executes the following stages:

Detection: The Ultralytics YOLOv8 detector (Ultralytics, version 8.4.37) [28] outputs a candidate set $C_{t}=\left\{1,\ldots ,N_{t}\right\}$, and each candidate contains a BBox, class label, and confidence score $c_{t,i}$.

Scoring: The semantic importance score $s_{t,i}$ is computed with AoI weighting, and per-mode utility $u_{t,i}\left(m\right)$ is further obtained with CSI awareness.

Scheduling: Under the instantaneous budget constraint, the system selects a transmission set $S_{t}$ consisting of BBox-only items and ROI patches.

Transmission: The selected content is transmitted through the channel model and reconstructed at the receiver.

Evaluation: Reconstructed patches are re-detected to obtain task-proxy metrics and resource-consumption statistics.

### 3.2. Budget and Unified Resource Accounting

To compare analog-style (JSCC/uncoded) and digital (JPEG) pipelines without bias, we adopt a fixed physical budget induced by the link bit rate and frame rate. Given a link budget *B* (kbps) and frame rate *f* (fps), the per-frame budget in bytes is

(1) $$B_{t}=\frac{B\times 1000}{8\times f}.$$

Under a fixed kbps/fps configuration, $B_{t}$ is constant across frames; we retain the subscript *t* only to keep the notation compatible with possible time-varying budgets.

To ensure a fair system-level comparison across coding regimes, we explicitly separate two notions:

- budget (bytes): The external per-frame budget $B_{t}$ induced by kbps/fps, identical for all schemes under the same link configuration;
- resource utilization: The fraction of the budget that is actually consumed by the scheduler, defined as $\mathrm{Util}=E\left[B_{t}^{\mathrm{used}}\right]/B_{t}$ at the dataset level, where $B_{t}^{\mathrm{used}}$ is the realized transmitted bytes per frame.

In all budget-sweep experiments, we report Util together with the chosen patch sizes and the task metrics. This directly addresses cross-scheme fairness using the same physical budget (kbps/fps) without assigning optimistic information-theoretic credits to any particular coding regime.

### 3.3. Channel and Coding Schemes

This paper adopts AWGN as a controllable baseline channel model and additionally performs a Rayleigh fading sanity check to reflect time-varying IoV links. For the digital baseline (JPEG), we optionally introduce non-ideal link behaviors (e.g., packet drops) to emulate no-retransmission scenarios. For JSCC and uncoded transmission, reconstruction is performed directly under the analog channel model. We emphasize that the BBox-only mode is a lightweight metadata fallback and does not constitute learned semantic communication by itself; the learned semantic communication component in this paper refers to the analog-style transmission of ROI patches (e.g., JSCC).

In our implementation, analog-style schemes occupy a fixed number of real channel uses determined by patch size and latent representation: uncoded transmission sends 3×p×p pixel values as real symbols, while JSCC sends a learned latent tensor as real symbols with power normalization. For the digital baseline (JPEG), we treat the compressed payload as the transmitted data and model link degradation via controlled drops or capacity-aware adaptation in robustness studies. Real IoV links are usually time-varying fading channels, such as Rayleigh or Rician channels, and are further affected by Doppler spread and shadowing. In this paper, AWGN is adopted as a controllable baseline, and time-varying channel conditions are abstracted through the frame-wise SNR $\gamma_{t}$, which can be interpreted as the effective post-equalization SNR reported by the receiver. This abstraction allows the paper to focus on the system-level closed loop of detection, scheduling, and transmission while preserving CSI-aware adaptation.

### 3.4. Task-Proxy Metrics

To mitigate the effects of class noise and annotation mismatch in cross-domain deployment, we adopt a class-agnostic task evaluation by default, i.e., class matching is ignored and a detection is counted as successful if the IoU criterion is satisfied. The system reports:

- Task-proxy detection success rate: The fraction of objects that remain detectable after reconstruction;
- Task-proxy mean IoU: The average IoU of reconstructed detections;
- Task-proxy confidence drop: The average decrease in detector confidence after reconstruction (smaller is better).

The proxy metrics used here are intended to reduce the influence of label-space mismatch and annotation noise in cross-domain comparison. For completeness, we also evaluate receiver-side detections against COCO-format annotations when available. To complement the proxy with a standard annotation-based metric, we also report COCO-style Average Recall with AR100 (max detections = 100) under ROI-only evaluation [29]. Because ROIs are selected by the same scheduler for both receiver reconstructions and their pre-channel oracle, we further report Tx-normalized AR100 (TxNorm-AR100), defined as the ratio between receiver AR100 and the oracle AR100 under the same transmitted ROIs, to isolate transmission-induced degradation from ROI-selection effects.

### 3.5. Notation

Key symbols used throughout the paper are summarized in Table 1.

## 4. Problem Formulation: Online Semantic Selection Under Budget Constraints

At each frame *t*, the system first obtains a candidate set $C_{t}=\left\{1,\ldots ,N_{t}\right\}$ from the object detector. Under limited and time-varying communication resources, the scheduler must determine the transmission action for each candidate $i\in C_{t}$.

We define the multi-scale transmission-mode set as M={0}∪P, where mode 0 denotes BBox-only transmission (lightweight metadata) and p∈P denotes ROI patch transmission at resolution *p* (task-relevant visual content). Let $x_{t,i}^{m}\in \left\{0,1\right\}$ be a binary decision variable indicating whether object *i* uses mode *m* at frame *t*. To avoid redundant resource allocation, each object can use at most one mode:

(2) $$\sum_{m\in M}x_{t,i}^{m}\leq 1,\;\forall i\in C_{t}.$$

The inequality rather than equality leaves room for a null action, i.e., the scheduler may choose not to transmit object *i* in frame *t*.

Each mode *m* incurs a communication cost $b_{t,i}\left(m\right)$ and yields a semantic utility $u_{t,i}\left(m\right)$. Accordingly, the total per-frame communication cost $C_{t}$ and total semantic utility $U_{t}$ can be written as

(3) $$\begin{array}{cc} C_{t} & =\sum_{i\in C_{t}}\sum_{m\in M}x_{t,i}^{m}\;b_{t,i}\left(m\right),\\ U_{t} & =\sum_{i\in C_{t}}\sum_{m\in M}x_{t,i}^{m}\;u_{t,i}\left(m\right). \end{array}$$

Later, when the selected set of object-mode pairs is written explicitly as $S_{t}$, we use the equivalent notation $C_{t}=C\left(S_{t}\right)$ and $U_{t}=U\left(S_{t}\right)$.

The global optimization problem is

(4) $$\begin{array}{cc} \left(P1\right):\max_{\left\{x_{t,i}^{m}\right\}}\; & \lim_{T\to \infty }\frac{1}{T}\sum_{t=1}^{T}E\left[U_{t}\right]\\ s.t.\; & \lim_{T\to \infty }\frac{1}{T}\sum_{t=1}^{T}E\left[C_{t}\right]\leq \lim_{T\to \infty }\frac{1}{T}\sum_{t=1}^{T}\rho B_{t},\\ \; & C_{t}\leq B_{t},\;\forall t,\\ \; & \sum_{m\in M}x_{t,i}^{m}\leq 1,\;\forall i\in C_{t},\forall t,\\ \; & x_{t,i}^{m}\in \left\{0,1\right\},\;\forall i\in C_{t},\forall m\in M,\forall t. \end{array}$$

Here, ρ∈(0,1] denotes the target budget ratio. Problem (P1) is a cross-frame extension of the Multiple Choice Knapsack Problem and exhibits strong temporal coupling together with non-convex combinatorial structure. Because future target dynamics and channel evolution are unknown in advance, direct global optimization is impractical.

## 5. Proposed Method: AoI/CSI-Aware Virtual-Queue Scheduling

### 5.1. AoI-Aware Importance Scoring

In the proposed system, semantic importance is decomposed into two layers: static semantics, represented by detection confidence and class prior, and temporal semantics, represented by freshness through AoI.

(1) Static semantic score

For object *i*, let $c_{t,i}\in \left[0,1\right]$ be the detector confidence and $k_{t,i}$ be the class label. The base semantic score is defined as

(5) $$s_{t,i}^{\mathrm{base}}=w\left(k_{t,i}\right)\cdot c_{t,i},$$

where w(·) denotes the class weight. In practice, higher weights can be assigned to pedestrians and two-wheelers to better reflect road-safety relevance [16,30].

(2) AoI tracking and freshness weighting

This paper employs a lightweight IoU tracker for cross-frame target association: objects of the same class are regarded as the same track if their IoU exceeds a threshold $\tau_{\mathrm{aoi}}$. The AoI age of every track increases by 1 at each frame, and is reset to 0 once the track is successfully transmitted. Let $a_{t,i}$ denote the current AoI age of object *i*. The AoI-weighted semantic score is then defined as

(6) $$s_{t,i}=s_{t,i}^{\mathrm{base}}\cdot \left(1+\beta \cdot f(a_{t,i})\right),$$

where β is the AoI weight and f(·) can take the form *a*, log(1+a), or a normalized variant. The experiments use f(a)=a by default. AoI is implemented on a per-frame basis and can be converted into seconds as $a_{t,i}/\mathrm{fps}$.

### 5.2. CSI-Aware Multi-Mode Patch Utility

The system uses a discrete set of patch modes P and explicitly models the relations between size and cost and between size and semantic gain.

(1) Mode set and cost

Each candidate can choose one of the following transmission modes:

- L0: BBox-only metadata, which has very low cost and remains available even at low SNR;
- L1/L2: patch mode *p*, whose cost increases with patch area.

We use an area-proportional cost model to capture that larger patches occupy more physical resources. In the system simulation, the realized cost is measured in transmitted bytes and depends on the codec and the link configuration; in the analysis below, we use a compact approximation that scales with patch area:

(7) $$b\left(m\right)=\left\{\begin{array}{cc} b_{0}, & m=0,\\ \lambda_{b}m^{2}, & m\in P, \end{array}\right.$$

and then write the object-wise resource cost as $b_{t,i}\left(m\right)=b\left(m\right)$. In our experiments, we use $\lambda_{b}=3$ to reflect RGB-channel scaling, while the exact realized byte consumption is recorded and reported in the budget-sweep fairness study (Section 6.6).

(2) Semantic gain from patch size

Larger patches typically contain more complete target appearance and contextual information, and are therefore assigned a gain term

(8) $$u^{\mathrm{gain}}\left(m\right)=\left\{\begin{array}{cc} u_{\mathrm{bbox}}^{\mathrm{gain}}, & m=0,\\ {\left(\frac{m}{p_{0}}\right)}^{\alpha }, & m\in P, \end{array}\right.$$

where $p_{0}$ is the reference patch size and α is the gain exponent.

(3) CSI-aware feasibility factor

Following recent adaptive-JSCC studies [11,12,13,14,15,16], we use a logistic gate to approximate the reliability of each mode:

(9) $$\phi_{m}\left(\gamma_{t}\right)=\sigma \;\left(\frac{\gamma_{t}-\tau_{m}}{\kappa_{m}}\right),\;\sigma \left(x\right)=\frac{1}{1+e^{-x}},$$

where $\tau_{m}$ is the SNR threshold and $\kappa_{m}$ is the smoothness parameter. For patch mode p∈P with base size $p_{0}=128$, we use

(10) $$\tau_{p}=6+4\left(\frac{p}{p_{0}}-1\right),\;\kappa_{p}=1.5.$$

The BBox-only mode m=0 uses a lower threshold and smoother slope so that it remains feasible under harsher channel conditions.

(4) Per-mode utility

For candidate *i* under mode *m*, the utility is defined as

(11) $$u_{t,i}\left(m\right)=s_{t,i}\cdot u^{\mathrm{gain}}\left(m\right)\cdot \phi_{m}\left(\gamma_{t}\right).$$

### 5.3. Virtual Queue and the DPP Objective

Using the action-set notation equivalent to $\left(C_{t},U_{t}\right)$ above, let $S_{t}$ denote the selected set of object-mode pairs at frame *t*, $C(S_{t})$ denote the actual resource cost, and

(12) $$U\left(S_{t}\right)=\sum_{\left(i,m\right)\in S_{t}}u_{t,i}\left(m\right).$$

The long-term stochastic optimization problem is

(13) $$\max\;\lim_{T\to \infty }\frac{1}{T}\sum_{t=1}^{T}E\left[U\left(S_{t}\right)\right]$$

subject to

(14) $$\lim_{T\to \infty }\frac{1}{T}\sum_{t=1}^{T}E\left[C\left(S_{t}\right)\right]\leq \lim_{T\to \infty }\frac{1}{T}\sum_{t=1}^{T}\rho B_{t},$$

with the instantaneous feasibility condition $C\left(S_{t}\right)\leq B_{t}$ for every frame *t*.

We define a virtual queue $Q_{t}$ that represents the accumulated budget deficit:

(15) $$Q_{t+1}=\max\left\{0,\;Q_{t}+C\left(S_{t}\right)-\rho B_{t}\right\}.$$

We further define the Lyapunov function $L\left(Q_{t}\right)=\frac{1}{2}Q_{t}^{2}$ and the one-step conditional drift $\Delta \left(Q_{t}\right)=E\left[L\left(Q_{t+1}\right)-L\left(Q_{t}\right)|Q_{t}\right]$. One can derive the drift upper bound

(16) $$\Delta \left(Q_{t}\right)\leq \Psi +Q_{t}E\left[C\left(S_{t}\right)-\rho B_{t}|Q_{t}\right],$$

where Ψ is a finite constant. Under the drift-plus-penalty framework,

(17) $$\Delta \left(Q_{t}\right)-VE\left[U\left(S_{t}\right)|Q_{t}\right]\leq \Psi +E\left[Q_{t}\left(C\left(S_{t}\right)-\rho B_{t}\right)-V\cdot U\left(S_{t}\right)|Q_{t}\right].$$

This leads to the single-slot maximization problem

(18) $$\max_{S_{t}}\;V\cdot U\left(S_{t}\right)-Q_{t}\cdot C\left(S_{t}\right).$$

#### Theoretical Guarantees and Performance Bounds

Under mild stochastic assumptions and standard boundedness conditions, the DPP policy enjoys the classical (O(1/V),O(V)) utility-queue trade-off [31]. Larger *V* generally yields better time-average utility but also increases the average virtual-queue length. Since the single-slot problem in this work is a discrete MCKP and we solve it with a greedy heuristic for real-time feasibility, strict single-slot optimality is not guaranteed; nevertheless, the virtual-queue mechanism still provides an interpretable cross-frame budget-stabilization effect.

### 5.4. Greedy Online Packing for MCKP

The single-slot objective is a Multiple Choice Knapsack Problem with an instantaneous budget $B_{t}$ and mutual-exclusion constraints. Because this problem is NP-hard and must be solved online, we use a two-stage greedy heuristic based on utility-cost density.

#### 5.4.1. Stage A: Local Mode Optimization

For each candidate *i*, we evaluate the DPP score under every feasible transmission mode m∈M with $b_{t,i}\left(m\right)\leq B_{t}$ and select

(19) $$m_{t,i}^{★}=\underset{m\in M:\;b_{t,i}\left(m\right)\leq B_{t}}{arg max}\left(V\cdot u_{t,i}\left(m\right)-Q_{t}\cdot b_{t,i}\left(m\right)\right).$$

We keep candidate *i* only when its best DPP score is strictly positive; otherwise, the candidate is discarded and the scheduler chooses the null action for that object.

#### 5.4.2. Stage B: Global Greedy Packing

For all retained candidates with a selected local mode, we compute

(20) $$\eta_{t,i}=\frac{V\cdot u_{t,i}\left(m_{t,i}^{★}\right)-Q_{t}\cdot b_{t,i}\left(m_{t,i}^{★}\right)}{b_{t,i}\left(m_{t,i}^{★}\right)},$$

sort candidates in descending order of $\eta_{t,i}$, and greedily pack them into $S_{t}$ while maintaining the accumulated cost not larger than the per-frame budget $B_{t}$. The packing process terminates once the next retained item would violate $C\left(S_{t}\right)\leq B_{t}$.

### 5.5. Complexity and Interpretability

Assume that the number of candidates is *N* and the number of patch modes is |P|. Local mode selection requires O(N|P|) operations, while sorting requires O(NlogN). The overall complexity is therefore O(N|P|+NlogN), which is sufficiently low for real-time execution on edge or roadside devices.

### 5.6. Algorithm Summary

The following pseudocode (Algorithm 1) summarizes the closed-loop logic of scoring, queueing, and packing for each frame.
**Algorithm 1:** Per-frame scoring–queueing–packing loop
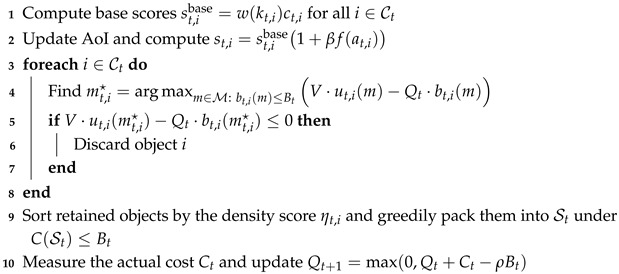


## 6. Experimental Evaluation

### 6.1. Ablations and Baselines

To verify the contribution of each component, we define the comparison mechanisms:

- No-AoI: set β=0, i.e., no extra weight is assigned to older objects.
- No-CSI: set $\phi_{m}\left(\gamma_{t}\right)\equiv 1$, i.e., patch size is no longer selected adaptively.
- No-Queue: set $Q_{t}\equiv 0$, i.e., no budget backpressure is imposed.
- No-BBox: remove the L0 BBox-only mode.
- BBox-only: allow only the BBox-only mode.
- Fixed-MaxPatch: always use the largest patch mode and never switch.

### 6.2. Experimental Setup and Benchmarks

To comprehensively validate the proposed dynamic scheduling strategy at the system level under resource-constrained IoV scenarios, we construct a complete end-to-end closed-loop simulation environment and carefully define the dataset preparation, model training, parameter settings, and baseline configurations.

The validation set consists of 10 driving clips from different road scenes. Each clip is approximately 30 s long, and around 200 consecutive frames are uniformly sampled at 10 fps. For reproducibility, the continuous clips are selected from the public BDD100K driving dataset [32] while preserving temporal continuity. Although the system motivation is multi-vehicle collaborative perception [33], the current experimental setup evaluates a single-stream closed loop (one video stream) to isolate the effects of scheduling and transmission under constrained budgets; explicit V2V coordination and bandwidth sharing among multiple vehicles are left for future work.

After the detector outputs candidate BBoxes, the system extracts transmission patches using the ROI cropping-and-resizing rule in Algorithm 2, which aligns patch scales, preserves contextual pixels via padding, and truncates at image boundaries without padding. The same rule is used to generate JSCC training patches and covers all patch scales used in the validation set.

Algorithm 2 provides the exact cropping rule used to extract an ROI patch around each detected object and resize it to the target patch resolution. This rule is applied consistently in both training (patch generation) and evaluation to avoid a train–test mismatch in the JSCC module.
**Algorithm 2:** ROI patch extraction and resizing
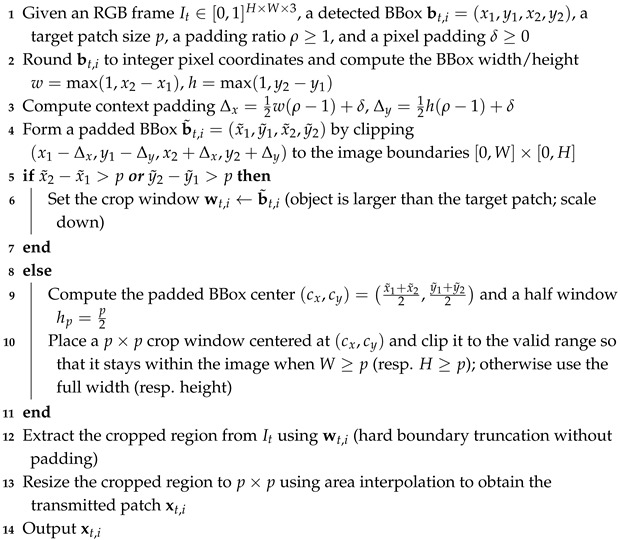


We adopt a minimal DeepJSCC-style convolutional autoencoder, summarized in Table 2. The encoder consists of three stride-2 convolutional downsampling blocks and one 3×3 convolution, producing a latent tensor with C=64 channels. Before transmission, the latent tensor is normalized to have approximately unit average power, and then transmitted over an AWGN channel at the current SNR. The decoder mirrors the encoder with deconvolution-based upsampling blocks and outputs an RGB patch in [0,1] via a sigmoid layer.

The training loss is pixel-wise MSE. During training, the SNR is uniformly sampled between 0 and 30 dB to improve robustness across channel conditions. Table 3 lists the key training settings.

Unless otherwise specified, the system frame rate is 10 fps and the maximum number of candidate targets processed in each frame is $K_{\mathrm{top}}=15$ (relaxed to 20 in the packing-strategy comparison). The Lyapunov drift-plus-penalty weight is V=6. For digital baselines (JPEG), we consider both an ideal-link setting (no explicit packet drops) and non-ideal-link robustness studies, where drop probabilities are scanned and applied symmetrically across coding regimes (Section 6.8).

The baselines and scan settings corresponding to the evaluation figures are summarized in Table 4.

### 6.3. Temporal Semantic Freshness (AoI) and System-Level Utility

**Figure 1:**
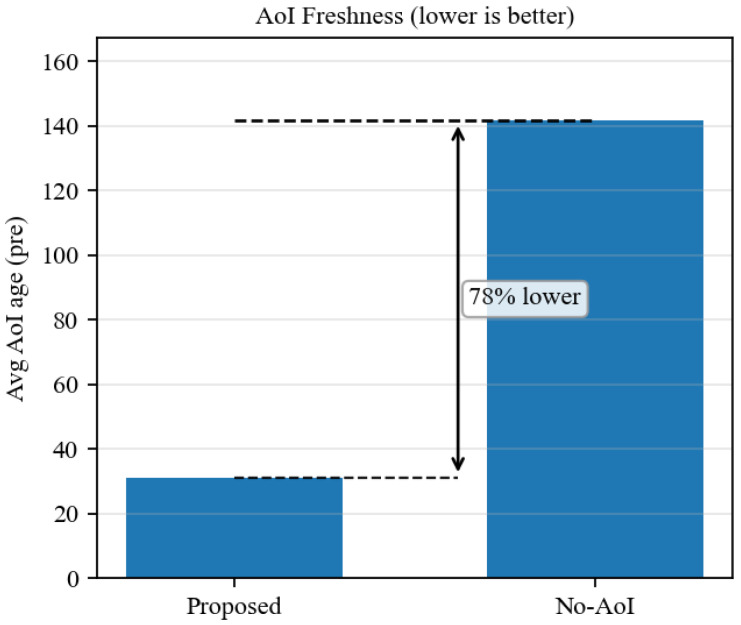
Effect of the Age of Information (AoI) mechanism on the average semantic staleness of the system.

Under the same budget, fixed SNR of 8 dB, and frame rate of 10 fps, we compare the proposed method with the No-AoI baseline. As shown in Figure 1, the No-AoI baseline repeatedly transmits the objects with the highest instantaneous confidence and yields a global average AoI age of 141.7. By contrast, the AoI-aware scheduler prioritizes long-unupdated critical objects and reduces the average semantic staleness to 31.2, corresponding to a reduction of approximately 78%.

**Figure 2:**
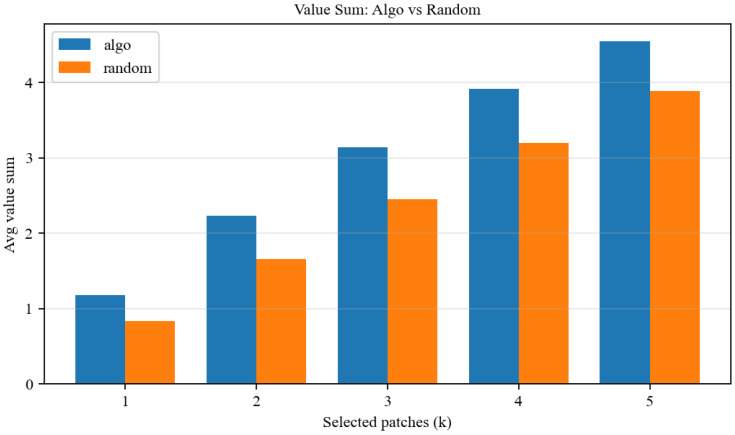
Comparison between value-prioritized packing and random selection in terms of average semantic value.

Under a fixed candidate truncation $K_{\mathrm{top}}=20$, we further scan the maximum number of transmitted patches allowed per frame, i.e., k∈[1,5]. Figure 2 shows that when only one target can be transmitted (k=1), the proposed approximate packing algorithm is equivalent to selecting the highest-value target. When multiple targets can be transmitted (k≥3), the value-density-based packing strategy better exploits the fragmented residual budget and achieves significantly higher cumulative semantic value than a random baseline.

### 6.4. Channel Adaptivity and the Resource–Semantics Trade-Off

**Figure 3:**
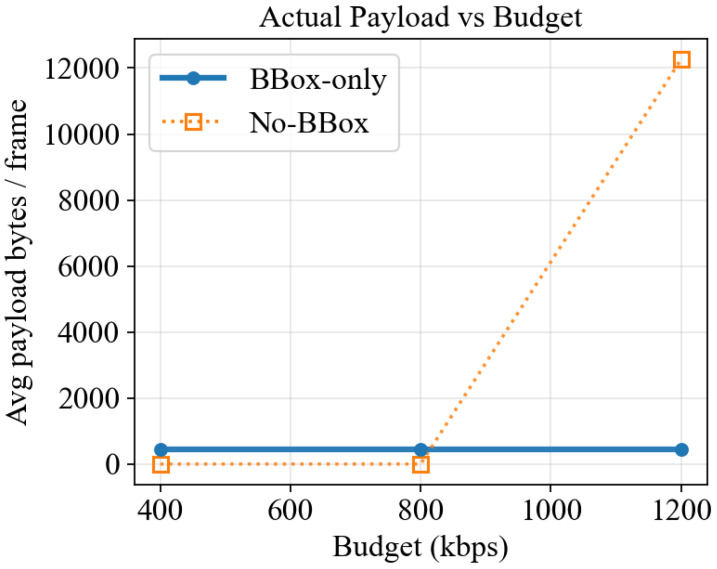
Average payload bytes per frame versus budget under extremely low rates (BBox-only vs. No-BBox).

We first examine the fallback role of the L0 metadata layer under extremely scarce bandwidth. Figure 3 plots the average payload bytes per frame versus the budget and shows that when the BBox-only mode is removed, the No-BBox baseline is unable to pack any patch at 400 or 800 kbps, leading to an empty transmission. Only at 1200 kbps does it barely transmit the smallest patch. By contrast, BBox-only remains feasible at all budget points with a stable overhead of around $4.5\times 10^{2}$ bytes.

**Figure 4:**
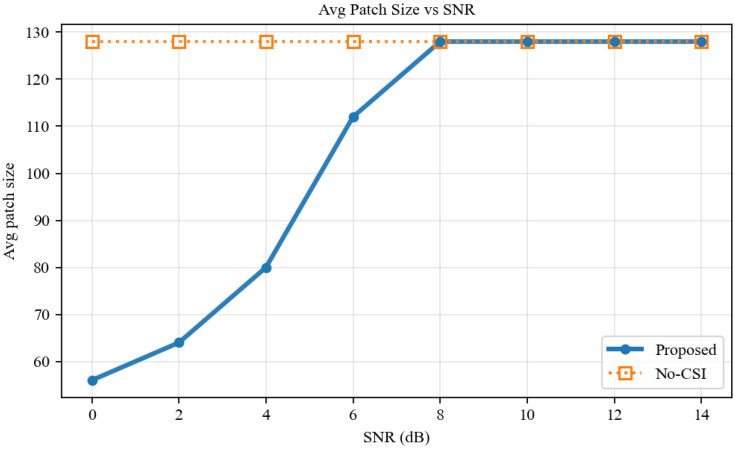
Adaptive evolution of the average transmitted patch size versus SNR.

Under a fixed per-frame physical budget induced by kbps/fps, we then scan discrete SNR points from 0 to 14 dB. As shown in Figure 4, as SNR increases, the scheduler exhibits a stepwise upgrade of patch size from 56 to 64, 80, 112, and 128 pixels. In contrast, the No-CSI baseline fails to adjust patch resolution in accordance with physical channel quality.

**Figure 5:**
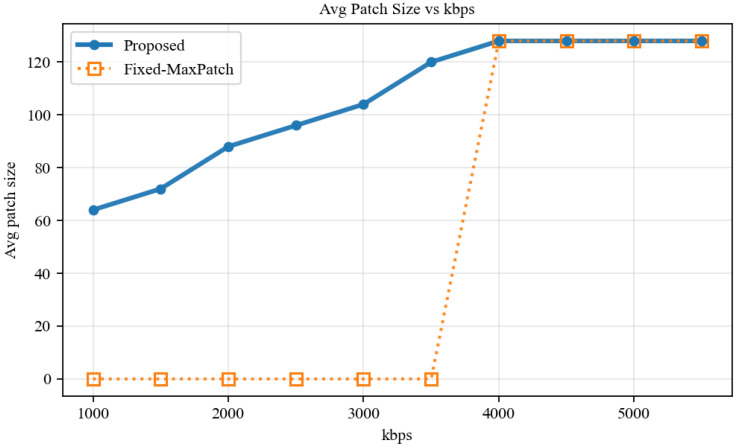
Adaptive evolution of the average transmitted patch size versus system budget.

Figure 5 further reveals the evolution of the multi-scale mechanism from the budget perspective. At a fixed SNR of 8 dB, as the available budget increases from 1000 to 5500 kbps, the scheduler steadily transitions from small patches to large patches, and can further degrade to BBox-only under even tighter budgets. By contrast, the Fixed-MaxPatch baseline remains essentially paralyzed until the budget exceeds the critical point around 4000 kbps.

### 6.5. SNR Sweep: JSCC Versus Uncoded Under a Fixed Patch Size

**Figure 6:**
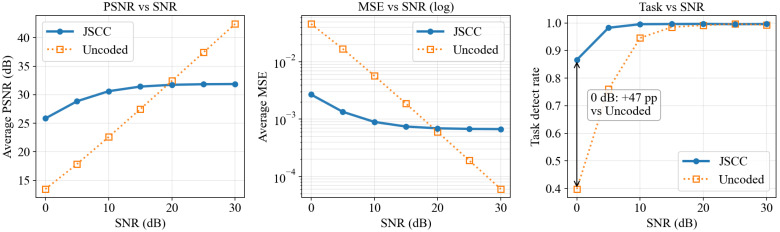
Reconstruction quality and task utility of JSCC versus uncoded transmission under an SNR sweep.

As shown in Figure 6, under a fixed budget and fixed patch size p=128, we scan the SNR range from 0 to 30 dB and compare JSCC with the uncoded baseline in terms of both reconstruction and task metrics. On the conventional image-quality metric PSNR, JSCC and Uncoded exhibit a crossover in the high-SNR region. The reason is that uncoded transmission approaches nearly lossless raw transmission under ideal channels, whereas JSCC is constrained by model capacity and exhibits a performance plateau. Mathematically,

(21) $$\begin{array}{cc} {\mathrm{PSNR}}_{i} & =10\log_{10}\frac{I_{\max}^{2}}{{\mathrm{MSE}}_{i}},\;I_{\max}=1,\\ E[\mathrm{PSNR}] & \neq 10\log_{10}\frac{1}{E[\mathrm{MSE}]} \end{array}$$

which explains why average PSNR alone may be misleading. The MSE subplot confirms that JSCC is substantially better in the low- and medium-SNR regimes, and the task-proxy detection-rate curve shows that JSCC remains consistently superior over the entire SNR range. In particular, at 0 dB, JSCC achieves a detection rate of approximately 0.866, while the uncoded baseline reaches only about 0.398.

### 6.6. System-Level Budget Sweep Under Low SNR

**Figure 7:**
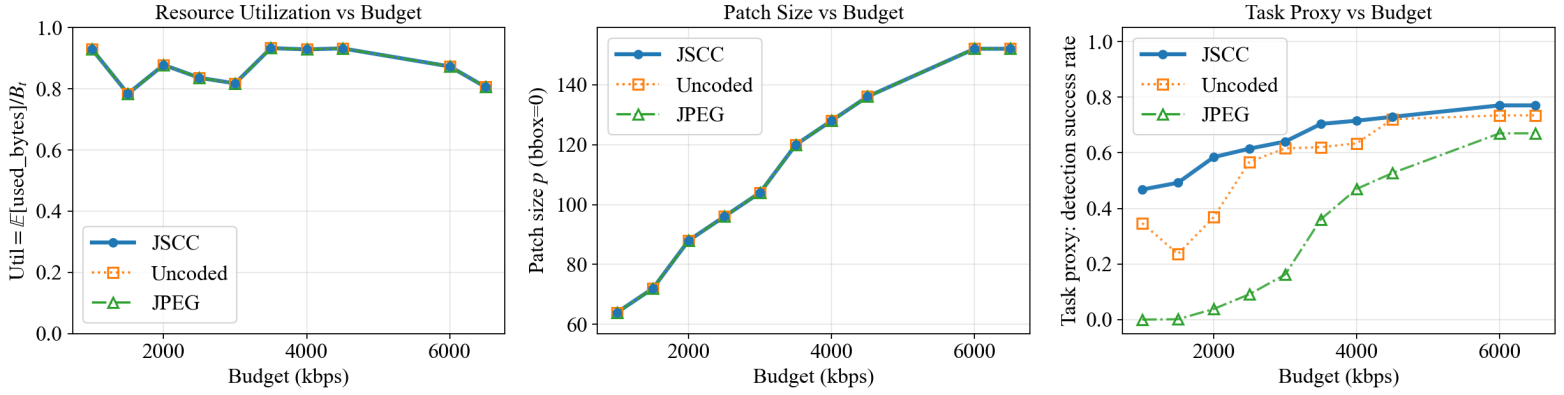
End-to-end budget sweep at SNR = 8 dB: (**left**) resource utilization $E\left[B_{t}^{\mathrm{used}}\right]/B_{t}$, (**middle**) selected patch size (auto-scaled), and (**right**) task-proxy detection rate after reconstruction (JSCC vs. uncoded vs. JPEG with capacity-aware adaptation and outage when the budget is insufficient).

We next evaluate the end-to-end system trade-off between resource utilization, selected patch resolution, and task utility under a fixed low-SNR condition (SNR = 8 dB). Figure 7 reports a three-panel comparison between JSCC, uncoded transmission, and a JPEG baseline, where all methods share the same external per-frame budget induced by kbps/fps. The left panel plots the realized budget utilization $E\left[B_{t}^{\mathrm{used}}\right]/B_{t}$, showing that the methods consume comparable resources under the same budget. The middle panel reports the selected patch sizes (auto-scaled), and the right panel reports the lightweight task-proxy detection success rate on reconstructed patches. The key observation is that, under tight budgets, JSCC maintains higher usable task performance without an abrupt collapse, whereas the digital baseline is more vulnerable to budget scarcity. In more intuitive terms, the left panel answers “did each method actually use the same physical budget?” and prevents an unfair comparison caused by hidden overhead or under-utilization. The middle panel shows how the scheduler trades patch fidelity for coverage as budgets change: when the budget is small, it prefers smaller patches so that at least some targets can be updated; when the budget increases, it upgrades patch size to improve reconstruction and task utility. The right panel then reflects the net effect on downstream usability: analog-style JSCC tends to degrade gracefully under tight budgets and noisy links, whereas separation-based digital coding may exhibit a more threshold-like behavior when the available budget is insufficient to deliver even the lowest admissible quality (thereby effectively dropping the ROI patch).

### 6.7. Robustness Versus SNR Under a Fixed Resource Budget

**Figure 8:**
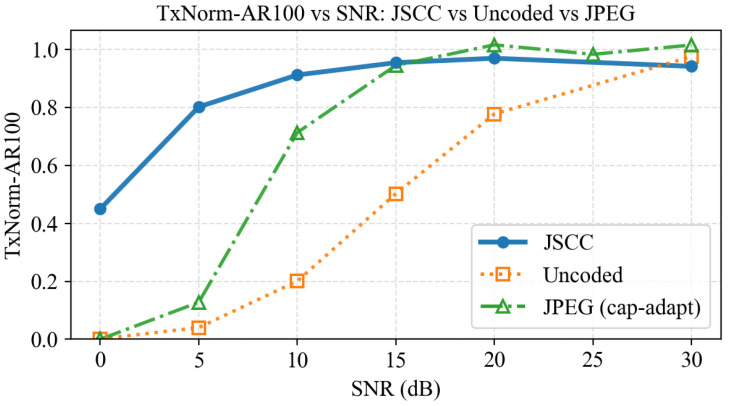
TxNorm-AR100 versus SNR (AWGN): JSCC vs. uncoded vs. JPEG (capacity-adaptive).

To assess robustness under varying channel quality, we scan discrete SNR points and report COCO-style AR100 under ROI-only evaluation. We further report TxNorm-AR100 to quantify the relative degradation between receiver reconstructions and their pre-channel oracle under identical transmitted ROIs. Figure 8 summarizes the SNR sweep for JSCC, uncoded transmission, and a capacity-adaptive JPEG baseline (which adapts quality to fit the same physical resource budget and drops when adaptation is insufficient). The results demonstrate that JSCC provides consistently higher task robustness at low SNR, while digital baselines exhibit a more pronounced “threshold” behavior under harsh channel conditions. Concretely, the capacity-adaptive digital baseline selects the highest JPEG quality whose compressed payload fits the deliverable budget under the current link condition; if no quality in the grid can be delivered within the same physical resources, the transmission is treated as an outage (a “cliff”), and the corresponding ROI is regarded as dropped for evaluation.

### 6.8. Symmetric Drop Sensitivity Scan

**Figure 9:**
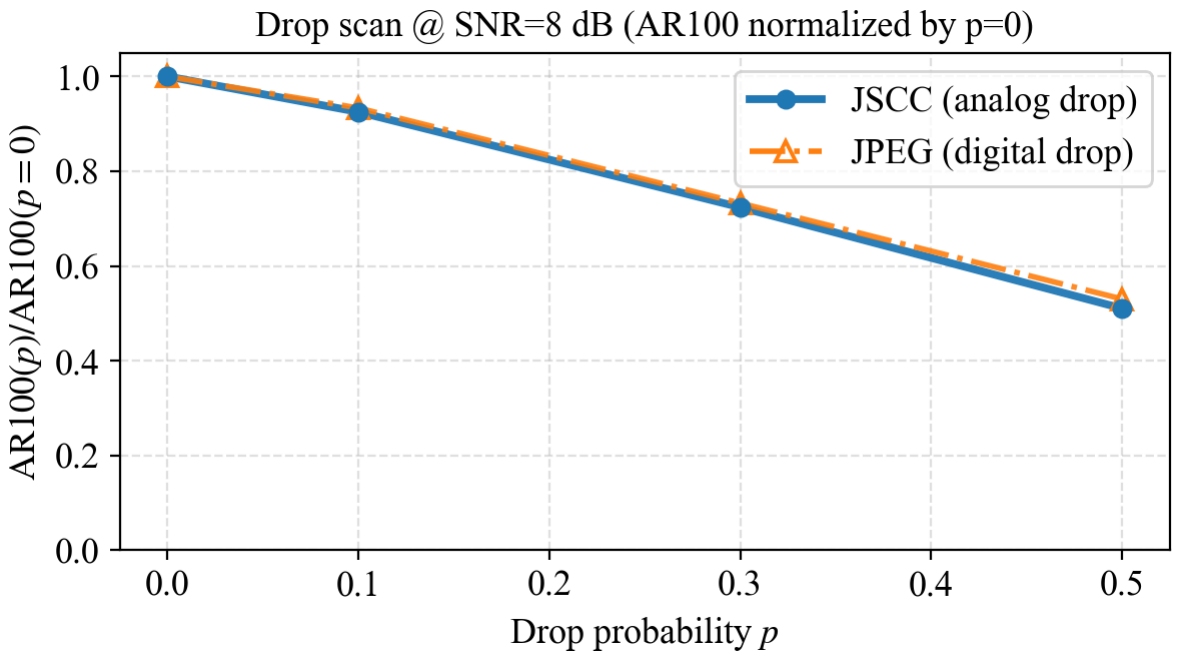
Normalized drop scan at SNR = 8 dB: AR100(p)/AR100(p=0) under symmetric drop modeling.

To avoid an arbitrary choice of a single packet-drop probability and to ensure symmetry across coding regimes, we scan a set of drop probabilities and plot the normalized task metric AR100(p)/AR100(p=0). For the analog-style scheme (JSCC), we apply analog erasures; for the digital baseline (JPEG), we apply i.i.d. packet drops. Figure 9 shows that the normalized degradation curves provide a fair comparison of sensitivity to drops under the same high-level erasure rate.

### 6.9. Fading Sanity Check

**Figure 10:**
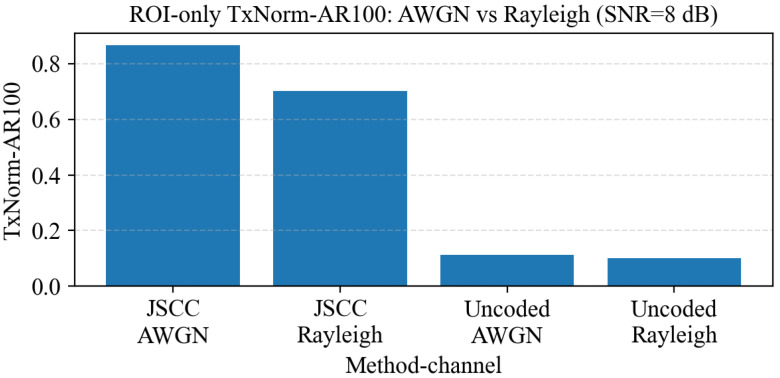
Fading sanity check at SNR = 8 dB: TxNorm-AR100 under AWGN versus Rayleigh.

Real IoV links often exhibit fading. As a minimal sanity check beyond AWGN, we compare the same configuration under AWGN and Rayleigh channels at the same reference SNR. Figure 10 reports TxNorm-AR100 for JSCC and uncoded transmission, showing that the relative task robustness trends remain consistent under fading.

### 6.10. Runtime Profile and Real-Time Feasibility

To support claims of real-time feasibility, we provide an end-to-end latency breakdown (ms/frame) covering detection, scheduling, and transmission-side processing. The profiling is conducted on a laptop equipped with an Intel Core i7-12650H CPU (Intel Corporation, Santa Clara, CA, USA), 32 GB RAM, and an NVIDIA GeForce RTX 4060 Laptop GPU (8 GB VRAM; NVIDIA Corporation, Santa Clara, CA, USA), running Microsoft Windows 11 (Microsoft Corporation, Redmond, WA, USA). Table 5 reports the breakdown.

## 7. Discussion

The experimental results suggest that closing the loop between perception-driven ROI selection and channel-aware online scheduling is beneficial under realistic resource constraints. In particular, the AoI/CSI-aware multi-mode policy enables smoother task-level degradation under tight budgets and adverse channel conditions. The resource-utilization analysis further indicates that the observed gains are not due to consuming more physical resources under the same kbps/fps budget, but are primarily achieved through more effective selection and mode switching. Together with the robustness scans (SNR sweep, drop scan, and fading sanity check), these results support the use of task-oriented, Tx-normalized metrics for evaluating end-to-end semantic communication systems.

## 8. Conclusions

This paper proposes a unified framework that integrates hierarchical transmission and Lyapunov-optimized online scheduling to enable task-oriented perception information delivery in IoV. The core idea is to shift from bit-level fidelity to task-level utility by jointly optimizing semantic extraction, mode selection, and online packing within a closed-loop system. By combining AoI/CSI-aware multi-mode patch scheduling with a virtual-queue budget-stabilization mechanism, the system performs more robust semantic selection under tight bandwidth constraints and time-varying channels. Under fixed kbps/fps budgets, the results demonstrate improved low-budget usability and smoother degradation under low SNR and unreliable links compared with digital baselines, while maintaining competitive performance as channel quality improves. The current study adopts a controllable end-to-end simulation to isolate the effects of hierarchical ROI transmission and online scheduling, and evaluates a single-stream closed loop rather than an explicit multi-vehicle bandwidth-sharing scenario. In addition, the physical link is abstracted by SNR-controlled AWGN/Rayleigh models, while the digital pipeline is emulated via capacity-aware adaptation and outage without explicitly modeling FEC/ARQ/MAC delays. Although we discuss representative semantic-communication and ROI-guided schemes from the literature, we do not claim a comprehensive head-to-head reproduction of all recent frameworks due to differences in tasks/datasets and incomplete public implementation details. Extending the evaluation to explicit multi-vehicle collaboration, richer channel and protocol dynamics, broader baseline coverage, and a wider range of embedded platforms remains an important direction for future investigation.

## Figures and Tables

**Table 1:** Notation and key symbols.

Symbol	Meaning
*t*	frame/time index
$C_{t}$	candidate set in frame *t*
$N_{t}$	number of candidates
$c_{t,i}$	detection confidence of object *i*
$a_{t,i}$	AoI age of object *i*
$\tau_{\mathrm{aoi}}$	IoU threshold for track association
M	transmission-mode set {0}∪P
$x_{t,i}^{m}$	mode-selection decision variable
$S_{t}$	selected set of object-mode pairs (action set)
$b_{t,i}\left(m\right)$	resource cost of choosing mode *m* for object *i*
$u_{t,i}\left(m\right)$	semantic utility of choosing mode *m* for object *i*
β	AoI weight
$\gamma_{t}$	channel SNR of frame *t* in dB
$\phi_{m}\left(\gamma_{t}\right)$	CSI-aware gating factor for mode *m*
$\tau_{m},\kappa_{m}$	logistic gate threshold and slope
P	patch-size mode set
$B_{t}$	per-frame budget (bytes/frame)
$B_{t}^{\mathrm{used}}$	realized transmitted bytes in frame *t*
Util	resource utilization $E\left[B_{t}^{\mathrm{used}}\right]/B_{t}$
$\lambda_{b}$	cost coefficient for BBox metadata (mode 0)
$C_{t}$	actual per-frame cost
$Q_{t}$	virtual-queue length
ρ	budget target ratio
*V*	DPP/Lyapunov weight

**Table 2:** JSCC patch autoencoder architecture used in our experiments.

Module	Layers (In Order)	Output Channels
Encoder	Conv 5×5, stride 2 + ReLU; Conv 5×5, stride 2 + ReLU; Conv 5×5, stride 2 + ReLU; Conv 3×3, stride 1	32→64→128→C
Channel	Power normalization; AWGN at SNR $\gamma_{t}$	*C*
Decoder	Conv 3×3, stride 1 + ReLU; Transposed-Conv 4×4, stride 2 + ReLU; Transposed-Conv 4×4, stride 2 + ReLU; Transposed-Conv 4×4, stride 2 + Sigmoid	128→64→32→3

**Table 3:** JSCC training protocol (key settings).

Item	Setting
Training samples	ROI patches extracted from large-scale driving images using Algorithm 2; resized to the target patch scale
Patch scales	Multi-scale training covering the patch-size mode set used in evaluation
Latent width	C=64 channels
Channel model	AWGN
SNR sampling	Uniform in [0,30] dB
Loss	Mean-squared error (MSE)
Optimizer	Adam
Batch size	32
Learning rate	$1\times 10^{-3}$
Epochs	30

**Table 4:** Overview of the baselines and scan settings for the evaluation figures.

Figure	Purpose	Baseline/Scheme	Scan Dimension
1	AoI freshness	Proposed vs. No-AoI	Fixed SNR = 8 dB; fixed budget (e.g., 2000 kbps)
2	Packing superiority	Algo vs. Random	*k*: 1–5; $K_{\mathrm{top}}=20$
3	BBox-only fallback	BBox-only vs. No-BBox	400/800/1200 kbps
4	CSI adaptation	Proposed vs. No-CSI	SNR: 0–14 dB; fixed budget
5	Multi-mode switching	Proposed vs. Fixed-MaxPatch	1000–5500 kbps; fixed SNR = 8 dB
6	SNR sweep	JSCC vs. Uncoded	SNR: 0–30 dB; fixed budget and p=128
7	System budget sweep	JSCC/Uncoded/JPEG	SNR = 8 dB; kbps/fps induces $B_{t}$; report $E\left[B_{t}^{\mathrm{used}}\right]/B_{t}$
8	Robustness versus SNR	JSCC/Uncoded/JPEG (capacity-adaptive)	SNR: 0–30 dB; fixed budget; metric: TxNorm-AR100
9	Drop sensitivity scan	JSCC (analog erasure) vs. JPEG (digital drop)	Scan drop probability *p*; metric: AR100(*p*)/AR100(0)
10	Fading sanity check	JSCC/Uncoded	AWGN vs. Rayleigh at SNR = 8 dB

**Table 5:** Latency breakdown on the 1k-sample set (ms/frame).

Stage	ms/Frame
Object detection	22.42
Online scheduling and packing	9.10
Transmission and reconstruction	12.23
Other processing overhead	9.59
Total	53.34

## Data Availability

Data and additional implementation details (e.g., run commands and configuration files) will be made available from the corresponding author upon reasonable request, subject to the dataset licenses and institutional policies.
